# 

**Published:** 1871-03

**Authors:** 


VOL. XXV. No. 3.

### THE Dental Register.

EDITED BY

J. TAFT & G. WATT.

MARCH, 1871.

MONTHLY:

THREE DOLLARS PER ANNUM, IN ADVANCE.

CINCINNATI:

WRIGHTSON & CO., Printers, 167 WALNUT STREET.

*J. & WM. TAFT, Dentists, 117 West Fourth St.*

